# Supplementary material for: Intraoral Scanners for In Vivo 3D Imaging of the Gingiva and the Alveolar Process
Source: J Clin Med. 2022 Oct 28;11(21):6389. doi: 10.3390/jcm11216389 (PMC9655054; doi:10.3390/jcm11216389)
Supplement: Supplementary file 1 [file jcm-11-06389-s001.zip › jcm-1951698-supplementary.pdf]

Intraoral scanners for in vivo 3D imaging of the gingiva and the alveolar process

Jonas Winkler , Anton Sculean and Nikolaos Gkantidis

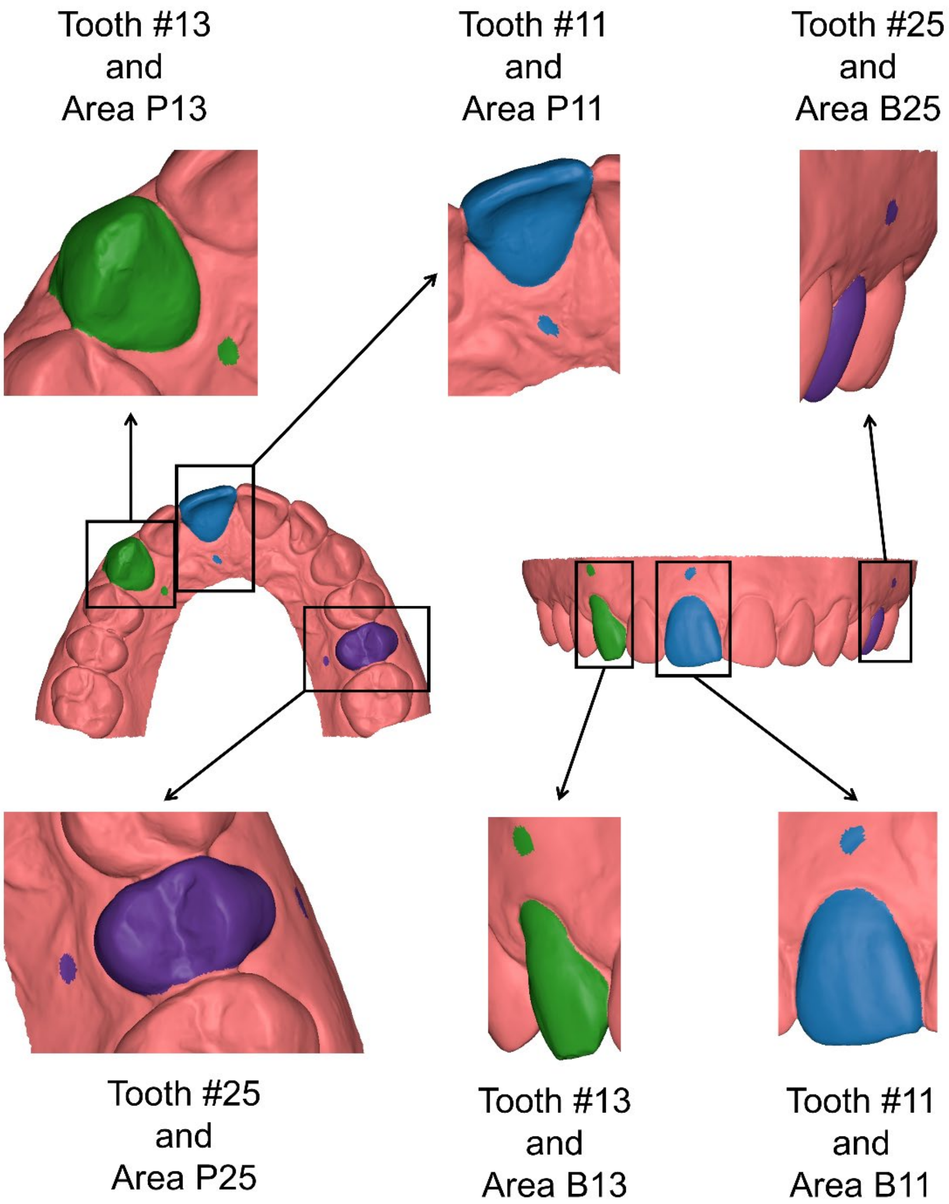

Figure S1. Superimposition areas and regions of interest (ROI) for single tooth measurements. Superimposition areas: clinical crown of tooth #11 (blue), #13 (green) and #25 (violet). ROIs: circular areas of approximately 2 mm diameter, centered 2 mm apically to the deepest point of the gingival margins of teeth #11, #13 and #25, on the buccal (B11, B13, B25) and palatal side (P11, P13, P25).

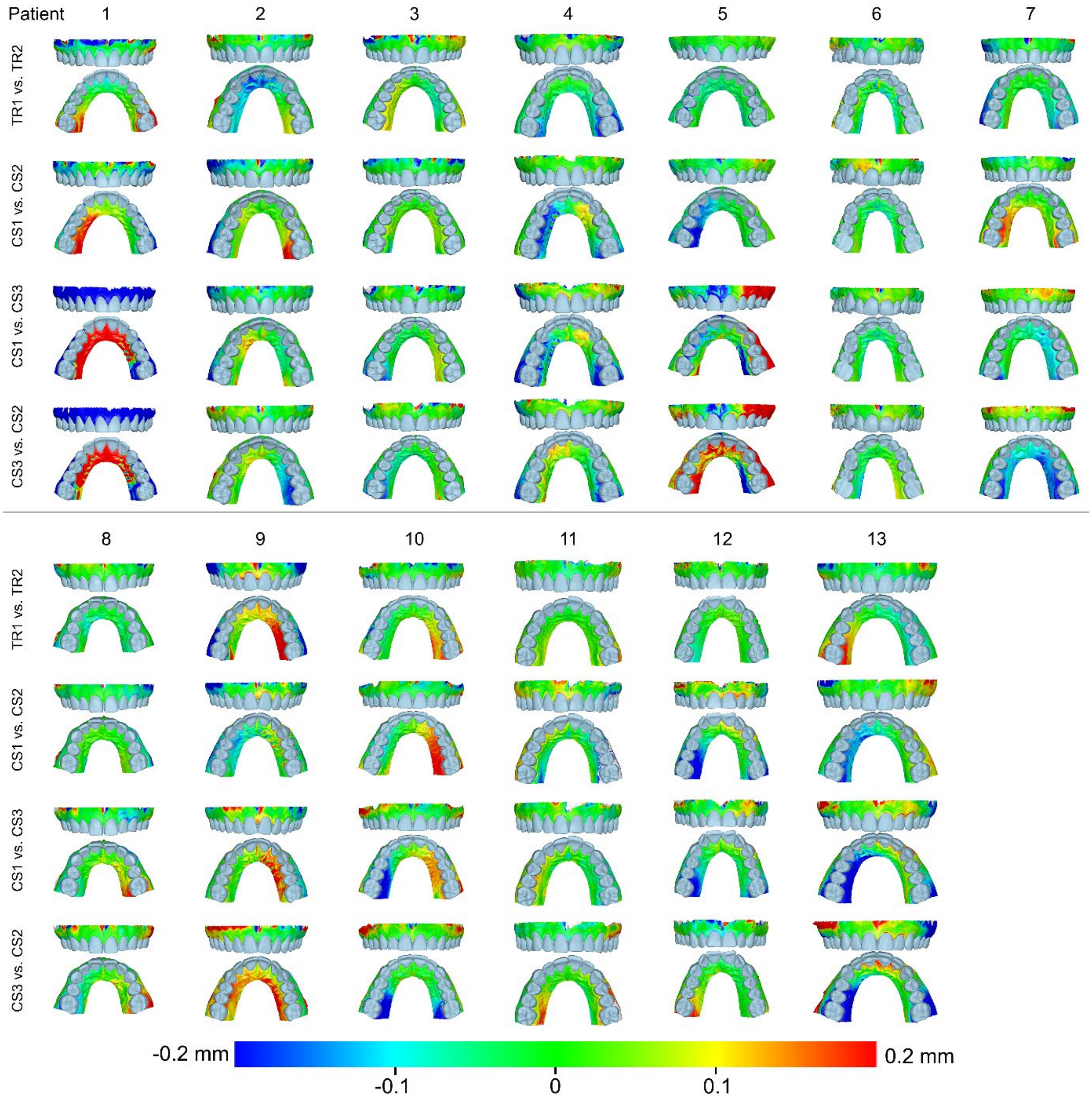

Figure S2. Colour-coded distance maps showing the precision of the intraoral scans at the buccal and palatal alveolar soft-tissue surfaces when superimposed on the buccal surfaces of the front teeth. Three scans from CS 3600 (CS1, CS2, CS3) and two scans from TRIOS3 (TR1, TR2) were assessed.

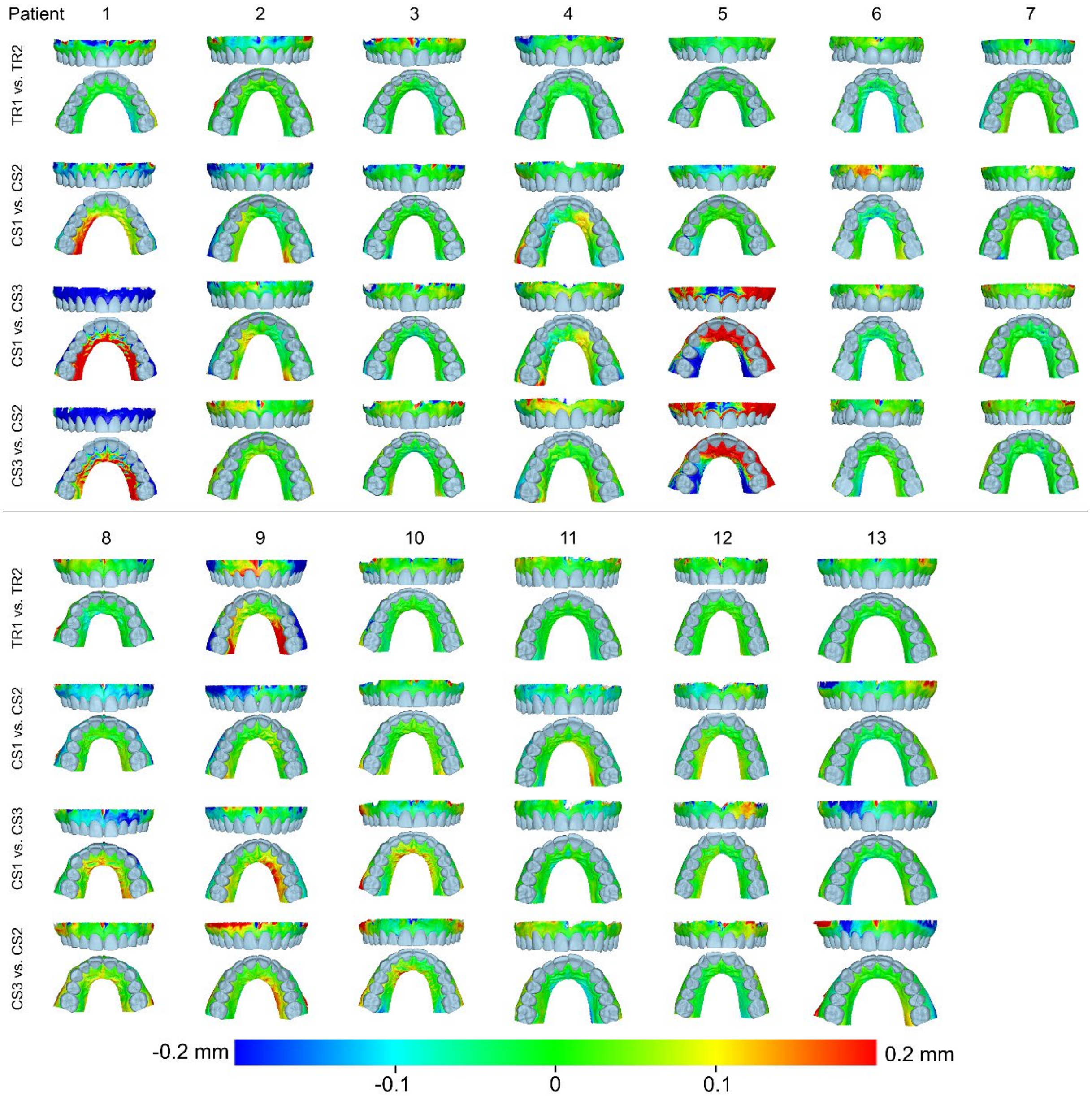

Figure S3. Colour-coded distance maps showing the precision of the intraoral scans at the buccal and palatal alveolar soft-tissue surfaces when superimposed on the dental arch. Three scans from CS 3600 (CS1, CS2, CS3) and two scans from TRIOS3 (TR1, TR2) were assessed.

Table S1. Pairwise comparisons of the precision of scanners in the imaging of the alveolar process detected through repeated scan superimpositions on different areas.

Superimposition area	Region of interest	P-value[1]		CS1vsCS3 - CS1vsCS2	CS2vsCS3 - CS1vsCS2	TR1vsTR2 - CS1vsCS2	CS2vsCS3 - CS1vsCS3	TR1vsTR2 - CS1vsCS3	TR1vsTR2 - CS2vsCS3
Alveolar process	Alveolar process	0.006	P-value[2]	0.507	0.075	0.064	0.221	0.039	0.019
Buccal front teeth	Alveolar process	0.109	P-value[2]	0.279	0.133	0.133	0.173	0.064	0.055
Dental arch	Alveolar process	0.016	P-value[2]	0.249	0.279	0.046	0.650	0.028	0.039

[1]Friedmann test, Level of significance: $p < 0.05$

[2]Wilcoxon signed-rank test (2-tailed), Level of significance following Bonferroni correction: $p < 0.01$

Table S2. Pairwise comparisons of the precision of scanners in the imaging of specific alveolar areas after superimposition on single teeth.

Superimposition area	Region of interest*	P-value[1]		B11_CS1vsCS3 - B11_CS1vsCS2	B11_CS2vsCS3 - B11_CS1vsCS2	B11_TR1vsTR2 - B11_CS1vsCS2	B11_CS2vsCS3 - B11_CS1vsCS3	B11_TR1vsTR2 - B11_CS1vsCS3	B11_TR1vsTR2 - B11_CS2vsCS3
#11	B11	0.048	P-value[2]	0.064	0.382	0.152	0.116	0.019	0.087
	P11	0.114	P-value[2]	-	-	-	-	-	-
#13	B13	0.378	P-value[2]	-	-	-	-	-	-
	P13	0.809	P-value[2]	-	-	-	-	-	-
#25	B25	0.595	P-value[2]	-	-	-	-	-	-
	P25	0.291	P-value[2]	-	-	-	-	-	-

*Circular areas of approximately 2 mm diameter, centered 2 mm apically to the deepest point of the gingival margin of teeth #11, #13 and #25, on the buccal (B11, B13, B25) and palatal side (P11, P13, P25).

[1]Friedmann test, Level of significance: $p < 0.05$

[2]Wilcoxon signed-rank test (2-tailed), Level of significance following Bonferroni correction: $p < 0.01$
